# Cyber Threat Intelligence-Based Malicious URL Detection Model Using Ensemble Learning

**DOI:** 10.3390/s22093373

**Published:** 2022-04-28

**Authors:** Fuad A. Ghaleb, Mohammed Alsaedi, Faisal Saeed, Jawad Ahmad, Mohammed Alasli

**Affiliations:** 1 School of Computing, Faculty of Engineering, Universiti Teknologi Malaysia, Johor Bahru 81310, Malaysia; 2 College of Computer Science and Engineering, Taibah University, P.O. Box 344, Medina 41411, Saudi Arabia; masadi@taibahu.edu.sa (M.A.); fsaeed@taibahu.edu.sa (F.S.); masali@taibahu.edu.sa (M.A.); 3 DAAI Research Group, Department of Computing and Data Science, School of Computing and Digital Technology, Birmingham City University, Birmingham B4 7XG, UK; 4 School of Computing, Edinburgh Napier University, Edinburgh EH10 5DT, UK; j.ahmad@napier.ac.uk

**Keywords:** malicious URLs, cyber threat intelligence, ensemble learning, internet security, cybersecurity

## Abstract

Web applications have become ubiquitous for many business sectors due to their platform independence and low operation cost. Billions of users are visiting these applications to accomplish their daily tasks. However, many of these applications are either vulnerable to web defacement attacks or created and managed by hackers such as fraudulent and phishing websites. Detecting malicious websites is essential to prevent the spreading of malware and protect end-users from being victims. However, most existing solutions rely on extracting features from the website’s content which can be harmful to the detection machines themselves and subject to obfuscations. Detecting malicious Uniform Resource Locators (URLs) is safer and more efficient than content analysis. However, the detection of malicious URLs is still not well addressed due to insufficient features and inaccurate classification. This study aims at improving the detection accuracy of malicious URL detection by designing and developing a cyber threat intelligence-based malicious URL detection model using two-stage ensemble learning. The cyber threat intelligence-based features are extracted from web searches to improve detection accuracy. Cybersecurity analysts and users reports around the globe can provide important information regarding malicious websites. Therefore, cyber threat intelligence-based (CTI) features extracted from Google searches and Whois websites are used to improve detection performance. The study also proposed a two-stage ensemble learning model that combines the random forest (RF) algorithm for preclassification with multilayer perceptron (MLP) for final decision making. The trained MLP classifier has replaced the majority voting scheme of the three trained random forest classifiers for decision making. The probabilistic output of the weak classifiers of the random forest was aggregated and used as input for the MLP classifier for adequate classification. Results show that the extracted CTI-based features with the two-stage classification outperform other studies’ detection models. The proposed CTI-based detection model achieved a 7.8% accuracy improvement and 6.7% reduction in false-positive rates compared with the traditional URL-based model.

## 1. Introduction

Recently, the number of users surfing the Internet has increased exponentially. Due to the proliferation of mobile devices, ad hoc networks, smart sensors, and the Internet of Things technologies fueled by the imposed lockdown to mitigate the COVID-19 pandemic, the Internet has become an essential part of people’s daily lives and activities worldwide [1,2,3,4]. Most businesses shifted online due to the availability of reliable infrastructures such as cloud storage, cost-effective platforms, and a large target market. However, the Internet brings many cyber threats such as malware, spamming, phishing, financial fraud, information theft, and data sabotage [3,4,5,6]. Malicious websites are the primary attack vector that is used by cybercriminals to spread malware and archive attackers’ objectives [1]. A malicious website contains content that can be harmful such as malware or phishing attacks infecting the visitors’ smart devices with malware without user interaction, such as clicking or downloading, with the website.

According to [5], 18.5 million websites are infected by malware. Moreover, according to Google’s safe browsing report [6], there were two million phishing websites in September 2020, an increase of nearly 2800% compared with the number in September 2010. Attackers spread fake information and advertisements to attract users to visit malicious websites. Once a victim visits a malicious website, attackers use different strategies to infect users browsing devices with malicious payloads or deceive victims into interacting with the attackers for financial fraud or other types of attacks. Many harmful websites are not intended to be malicious by the developers. Attackers can exploit vulnerable websites to perform malicious intent. For example, an attacker can inject cross-site scripting into a vulnerable website to steal a visitor victim’s sensitive information or perform a phishing attack [7].

The problem with detecting malicious websites has been around since early 2004 [8,9,10,11,12,13,14,15,16,17,18]. Many solutions have been proposed to accurately detect these websites. These solutions can be divided into three categories by their source of investigation: URL-based [8,9,10,11,12,13,14,15,16], web content-based [19,20,21], and script-based [17,18]. URL-based detection is the most investigated approach followed by content-based detection, while little research has been investigated on script-based detection. URL-based detection is preferable because it is a proactive and safe approach for the detection machines as it can detect the malicious URLs before it is visited by the user. Moreover, detecting malicious URLs is more efficient for real-time detection and resource-constrained applications such as mobile and Internet of Things (IoT) devices.

Various techniques have been suggested to detect malicious websites and harmful content by extracting features from their URLs [15,16,22,23,24,25,26,27,28]. Most of these techniques rely on humans to derive the features [16,22,23,24,25,26] while few solutions used deep learning techniques to automate the features [15,27,28]. Many sets of features were extracted and used for the detection including host information features such as country name and host sponsor, domain features such as .com and .tk, and lexical features such as the number of dots in URL and URL length. However, the URL-based features are subject to manipulation by attackers and can be dynamically changed, and may be insufficient for effective representation. Attackers can use evasive techniques to bypass the security countermeasures. Accordingly, any features extracted from these URLs can be misleading as attackers can manipulate them to hide the malicious intent and malicious patterns of the website. Therefore, features that are out of attackers’ control will be beneficial for improving detection accuracy and reducing the false alarm rate.

The CTI feature can be used to enrich URL-based features to improve detection performance. Cybersecurity analysts, users’ experiences, and website reputations can be important sources of information. People usually share knowledge regarding malicious websites in discussion forums, social media, and news websites. Cyber threat intelligence can be safer, more efficient, and provide more accurate results than investigating the website content. This study designed and developed a malicious URL detection model that utilizes cyber threat intelligence-based features to improve classification performance. The proposed model, called the Cyber Threat Intelligence-based Malicious URL Detection model (CTI-MURLD), consists of three main components. The first component is for feature collection. Three types of features are extracted: URL-based features, Whois information-based features, and cyber threat intelligence features. The threat factors are researched using Google searches and Whois information. The second component contains data processing, including feature extraction, representation, and selection. N-gram is used for feature extraction, the Term Frequency-Inverse Document Frequency (TF-IDF) technique is used for feature representation, and mutual information (information gain) is used for feature selection. The third component is classification and decision making. The RF algorithm was used to train three ensemble classifiers. Each classifier was trained using different features. The probabilistic outputs of the decision tree classifiers in each forest were aggregated and used to train a multilayer perceptron-based classifier for decision making. The multilayer perceptron-based classifier could learn the hidden patterns that map the classifier’s output with the correct class of URLs. We hypothesize that the multilayer perceptron-based classifier can be more effective than the random forest classifiers’ three independent majority voting schemes. The results of the experiments were validated employing commonly used performance measures and benchmarked using a widely accepted dataset that contains benign and malicious URLs. Additionally, a comparison of related studies was carried out that shows the superiority of the proposed work. The results show a significant improvement in the proposed model’s performance compared with the state-of-the-art models. This study makes the following contributions:

1. A malicious URL-detection model based on CTI was designed and developed. Both the URL and web content are subject to obfuscation; an independent source of features that are outside of the attacker’s control was needed to strengthen the model’s performance. Thus, cyber threat intelligence-based features were extracted from a Google search and Whois information and used as new knowledge to train the proposed detection model.
2. The study designed and developed an ensemble learning-based model that combines three random forest-based predictors such as predetection and feature extractions with multilayer perceptron-based classifiers for the final decision. Three RF classifiers were trained using different feature dimensions extracted from URLs, Whois, and Google-based CTI. The three majority voting schemes that were used by the trained RF classifiers were replaced by the trained multilayer perceptron-based classifiers for accurate detection.
3. Several machine learning algorithms have been investigated, including deep learning techniques such as the convolutional neural network (CNN) model and sequential deep learning model, which were trained to distinguish between malicious and benign patterns. Results demonstrated that the cyber threat intelligence collected from Google improves the detection performance of malicious websites.

The remainder of the manuscript is organized as follows: Section 2 reviews the related work; Section 3 describes the proposed model; Section 4 explains the experimental design; Section 5 presents the results with a detailed discussion; Section 6 presents the conclusion and future work.

## 2. Related Work

For many decades, malicious URL detection has been a major concern for cybersecurity specialists [8,9,10,11,12,13,14]. Several solutions have been proposed to detect malicious URLs and protect users from being victims of an attack. These solutions can be categorized based on the type of detection into feature-based detection or blacklist-based detection [23]. In feature-based detection, the features that represent the URLs are extracted and automatically analyzed while blacklist-based detection relies on user reports and expert analysis. The centralized blacklist is the most widely used detection method in practice. The Internet Protocol (IP) address of the malicious website is stored in a database through matching detection. The feature-based detection can be further categorized into URL-based features or web content-based features. In the former, the features are extracted from the URL’s characters using N-gram techniques or derived directly from the URL (i.e., the length of the URL, whether it contains a file, the status, request protocol, IP, domain name, and registrar information). Meanwhile, in the latter, the features are crawled from the web content in terms of text, HTML code, and programs scripts. Detecting malicious URLs is crucial as many attackers spread malicious links to legitimate websites such as social media platforms and e-mails. Moreover, some malicious URLs are spread by downloading malware which can infect the detection machine during the crawling. Furthermore, detecting malicious URLs is more efficient and accurate than detecting web content due to the high similarity of some malicious web content with legitimate content, for example, phishing and fraudulent websites. Accordingly, this study focuses on reviewing URL-based detection solutions.

In [26], the authors proposed an improved malicious URL-detection model based on a two-stage distance-metric learning approach, namely singular value decomposition and linear programming for feature extraction. A set of 62 features were extracted from the URLs including information from Whois such as top-level domain names (TLDs), registrar information, lexical features such as the number of dots, keywords, and reputation-based features. A dataset consisting of 33,1622 URLs was collected from “PhishTank” and used to train three machine learning classifiers for the evaluation, namely K-nearest neighbor, support vector machine, and neural networks. Results showed that the improvement of the proposed feature extraction method was significant. However, the results showed that the false alarm (false positive) and misrate (false negative) were still high.

Rakesh and Muthurajkumar [22] modified the C4.5 algorithm to detect cross-site request forgery. Authors in [23] analyzed malicious URLs to extract common features regarding attacker behavior. A similarity matching technique was used to detect attackers’ habitual behavior. A small set of features were extracted from the URLs. Chiramdasu and Srivastava [16] proposed a malicious-URL-detection model using logistic regression. Three sets of features were extracted, host information features such as country name and host sponsor, domain features such as .com and .tk, and lexical features such as the number of dots in the URL and URL length. He and Li [24] focused on the class imbalance issue and then trained a model using XGBoost with cost-sensitive learning for detecting malicious URLs. A total of 28 features were extracted from the domain name, Whois information, geographic information, and suspicious words. Despite the results demonstrating that the proposed model outperformed related studies, the poor sensitivity achieved is the main limitation of this model. Authors in [29] proposed ensemble learning using a support vector machine (SVM) and a neural network to identify the command and control (C&C) server. The classifiers were trained based on features extracted from Whois and the DNS of domains of C&C servers. Another study [25] extracted 117 features from URL features, lexical features, domain name features, webpage source features, and short URL features. Then, various decision-tree-based learning algorithms were studied including J48 decision tree, simple CART, random forest (RF), random tree, ADTree, and REPTree for detecting malicious URLs. Results showed that the random forest-based classifier outperformed other constructed classifiers. In [30], two classifiers were trained using naïve Bayes and logistic regression. Different sets of features were extracted including lexical features and textual features represented by terms frequency/inverse documents frequency (TF-IDF). In their experiments, logistic regression outperformed the naïve Bayes algorithm.

The performance of various deep learning techniques in detecting malicious URLs was evaluated in [28]. The evaluated techniques included the recurrent neural network (RNN), identity-recurrent neural network (I-RNN), long short-term memory (LSTM), convolution neural network (CNN), and convolutional neural network-long short-term memory (CNN-LSTM). The model constructed using LSTM and the hybrid network of CNN and LSTM outperformed other studied models.

To summarize, many solutions have been proposed for detecting malicious URLs [16,22,26]. Most of these solutions utilize supervised-based machine learning techniques for classification [16,26,29]. The deep learning approach has also been investigated [28]. However, most of these solutions extract the features solely from URLs such as lexical features, textual features, and host features. It is commonly agreed among researchers that obfuscated URLs and web content hinder effective detection. CTI has not yet been investigated for improving detection performance. Therefore, this study proposed a malicious URL detection model that utilized CTI to extract features safely without crawling the actual malicious websites. User expertise regarding malicious URLs can be used for the early detection of URLs without the need for intensive analysis of the websites. Due to their classification performance and ability to extract effectiveness patterns from textual-based features, the RF algorithm and the multilayer perceptron were combined to improve the classification performance. A detailed description of the proposed model is provided in the following section.

## 3. The Proposed CTI-MURLD Model

**Figure 1:**
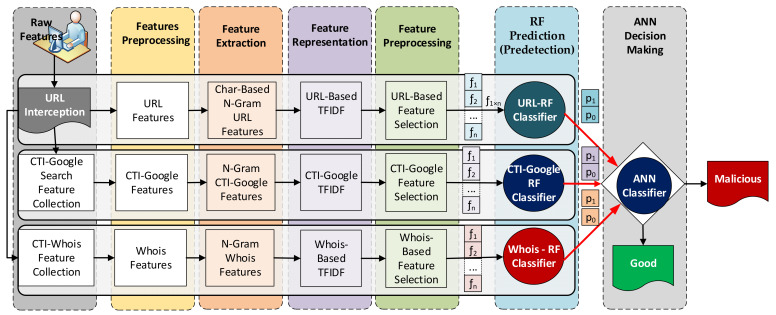
The proposed CTI-MURLD model.

Figure 1 shows the proposed Cyber Threat Intelligence-based Malicious URL Detection (CTI-MURLD) model. The proposed CTI-MURLD model consists of seven phases: data collection, feature preprocessing, feature extraction, feature representation, feature selection, ensemble learning-based prediction, and decision making. In the first six phases, three ensemble-learning-based predictors were constructed using the random forest (RF) algorithm. Each RF-based predictor was trained using different feature sets, URL-based, Google-based CTI, and Whois-based features. Each RF classifier had two probabilistic outputs. The first output represents the belief that a sample is a malicious URL ($p_{0}\;$ in Figure 1), and the second output is the amount of belief that the URL is benign ($p_{1}$ in Figure 1). In the last phase, an artificial neural network (ANN) classifier was built for decision making. The probabilistic outputs of the three RF classifiers were used to train the ANN classifier for the final decision. As shown in Figure 1, the URLs requested by users were intercepted, and three types of features were extracted. Each type of feature set was preprocessed to remove the noise. Then, more features were extracted using the N-gram technique and then represented by the TF-IDF technique. Then, the most representative features in each set were selected from each feature set (denoted by $f_{1}$ to $f_{n}$) using information gain. Each feature set is passed to its specifically trained RF predictor. The probabilistic outputs (two probabilistic outputs for each predictor) of these predictors were fed into the ANN classifier for the final decision about the URL class, whether it was malicious or benign. A detailed description of each phase is presented in the following subsections.

### 3.1. Phase 1: Data Collection Phase

In this phase, three types of features were collected, namely, URL content features, cyber threat intelligence data crawled using a web search (Google-based CTI), and data related to the domain owners crawled from Whois lookup (Whois-based CTI). The URL data were collected by intercepting user HTTP requests in the application layer. To collect the Google-based CTI, first, the domain name was extracted from the URL and the IP address of the domain was searched, and then data related to the domain and its IP were crawled from a Google search. The Whois-based CTI was crawled from a Whois search which included the website owner, creation date, contacts, domain status, registrant email, and registrant country.

### 3.2. Phase 2: Data Preprocessing

In this phase, the textual data that were collected in the previous phase were sanitized and normalized. The URL was preprocessed by removing symbols while the Google-based CTI and Whois-based CTI data were preprocessed using natural language processing (NLP) text-preprocessing techniques. Since Google-based CTI and Whois-based CTI data were crawled from websites, unwanted text such as HTML codes, symbols, and punctuation were removed to reduce feature complexity and enhance the classification performance. The collected text data were converted to lower case and then normalized. The normalization process aims were two-fold. Firstly, to convert the text from unstructured data to a structured word vector. Secondly, to reduce the scarcity of the feature vectors by removing unnecessary words and reducing the number of words by rooting the words to their originals. The normalization started with tokenization, then the removal of stop words, lemmatization, stemming, and finally converting the words to their equivalent numerical format. Tokenization is the process of representing the text sample by a list of words that construct the URL data sample. Stemming is converting the words into their roots e.g., removing “s” from the plural words and removing “ing” from the word. Lemmatization is the process of converting the words into base form by rooting the verbs to their root using lexical knowledge base e.g., ‘took’ to ‘take’.

### 3.3. Phase 3: N-Gram Feature Extraction 

The N-gram technique [31] was used to enrich the feature sets and create more representative features. N-gram has been a commonly used method for malicious URL detection and text analysis due to its effectiveness in improving the classification accuracy as reported by previous researchers [32,33,34,35,36]. Both word N-gram and character N-gram were used in this study. The character N-gram was used to extract features from the URL while the word N-gram was used for Google-based CTI and Whois-based CTI data. The URL data were converted to vectors of words each consisting of three, four, or five characters. To reduce feature complexity the word bi-gram technique was used for Google-based CTI and Whois-based CTI data. Each subsequent word was considered one additional feature. The output of this phase was three feature vectors each consisting of sets of words called tokens.

### 3.4. Phase 4: TF-IDF Feature Representations

To convert the words (the tokens) to their equivalent numerical values, a corpus that contained the list of unique tokens was constructed based on their frequency of occurrence in each class. Then, the statistical-based text representation, namely TF-IDF was calculated using the following equation:

(1) $$tf\_idf=tf.log\frac{N}{df}\;$$

where tf is the term frequency of the word in a specific instance, df is the document frequency for the word, N is the number of samples in the dataset. The term frequency tf is the number of times a word has occurred in the sample while the inverse document frequency idf refers to the inverse number of documents where the word has occurred. The higher the tf_idf of a word in a document, the more relevant the document. The output of this phase was three numerical vectors for each sample.

### 3.5. Phase 5: Feature Selections

In this phase, the features that represented the URL well were selected using information gain (mutual information). As the CTI features were collected from Google, a huge number of irrelevant features were included. These irrelevant features hindered the ability to differentiate between benign and malicious URLs due to the high dimensionality of the features. Thus, the learning task became complex, leading to poor training accuracy [28,37]. Similarly, the Whois information and URL features also contained irrelevant features, especially when the N-gram was used. The features were doubled based on the n-value of the N-gram. Moreover, feature selection is common research procedure for text-based features [12,28,37,38]. Therefore, feature selection is important in this study. However, this study selected the top five thousand features to minimize the probability of losing some information while maximizing the generalizability of the trained models.

The features with low probabilities (the uncommon features) have more information compared to features with high probabilities (the common features). The mutual information-based feature selection uses entropy to measure the impurity of the features when it is used to split the target variable. The entropy can be calculated using Equation (2). The higher the entropy the more information. Mathematically, the entropy is written as:

(2) $$E\left(p\right)=-\overset{n}{\underset{i=1}{\sum }}p_{i}\log\left(p_{i}\right)$$

where n is the target class, $p_{i}$ the probability of a feature split the class i. The information gain which represents the quality of the split can be calculated using the following equation.

(3) $$Gain=1-E\left(p\right)$$

where n is the target class of the entropy and the Gain is the quality of the split. A feature is important for classification if it has a high gain. The higher the gain, the lower the entropy. If the entropy is zero, the less impure the split. The output of this phase is a feature vector with only high-gain features selected.

### 3.6. Phase 6: RF Ensemble-Based Prediction

**Figure 2:**
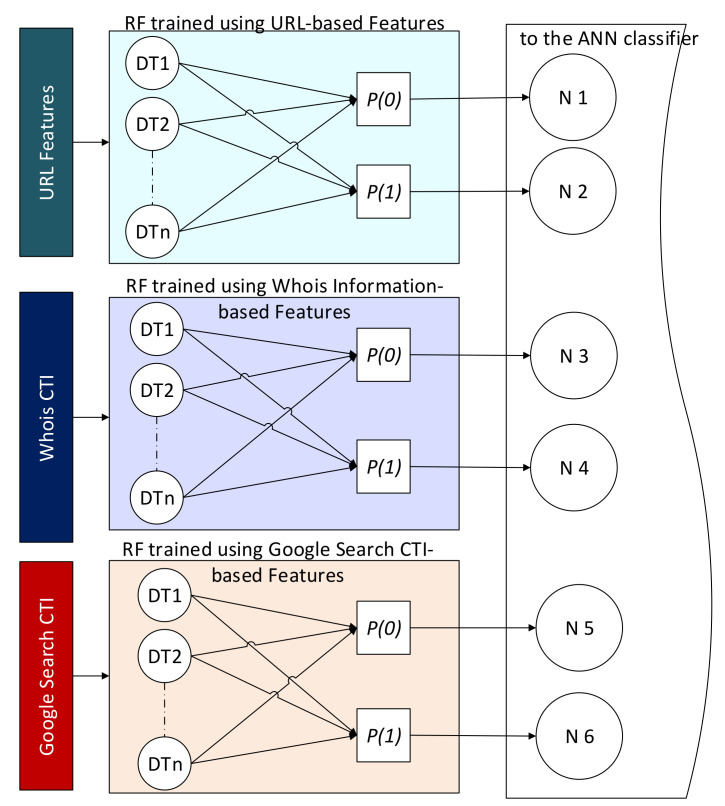
The new features extracted from the three ensemble models.

Three predictors were constructed and grouped using the RF algorithm in this phase. A predictor was trained for each type of feature, namely URL, Google-CTI, and Whois-CTI. A random forest algorithm was selected to construct these predictors. RF was selected for two reasons: firstly, for its diversity, which fits the diverse nature of the features in our collected datasets, and secondly for its effectiveness with high-dimensional data. Even after selecting a subset of important features, a high-dimensional vector consisting of 5000 elements was selected so that we did not lose the valuable features and generalizability. RF is a supervised machine learning algorithm that trains ensembles of weak classifiers using decision trees and bagging methods. The RF algorithm searches for the best split in a random subset of features before the tree is constructed. Thus, diverse trees were constructed that would improve the model’s performance. The RF classifier was constructed using 100 decision tree classifiers. Each decision tree classifier was trained based on a random subset of the original features with a random subset of the training dataset. The results were three forests of weak but diverse classifiers. The probabilistic outputs of these weak classifiers were averaged to be used as the RF decision about the class of the sample. The output of a tree was a real number between zero and one for each class. When the output value approaches zero, it means a low probability that the sample belongs to that class. Because the trees in the three RF classifiers were trained based on three different datasets, the results were more diverse and thus the probabilistic output. Instead of using the majority voting as the RF, in this study, the probabilistic outputs of the ensemble classifiers are used as input to the artificial neural network (ANN) classifier for decision making. Meanwhile, if the output value approaches one, it indicated a high belief that the sample belonged to that class. Figure 2 illustrates how these probabilities are extracted and fed as new features to the next stage of classification for decision making.

In Figure 2, $P\left(0\right)$ is the average probability of predicting a benign URL. In contrast, $P\left(1\right)\;$ is the average probability of predicting a malicious URL, DT denotes a decision tree (the weak classifier), and N1−6 represents the neuron node in the ANN model. These probabilistic values can be calculated as follows

(4) $$P\left(0\right)=\frac{\sum_{i=0}^{n}p\left(class\_label=0\right)}{n}\;$$

(5) $$P\left(1\right)=\frac{\sum_{i=0}^{n}p\left(class\_label=1\right)}{n}$$

where n denotes the total number of the estimators in each forest. These outputs are aggregated using a voting scheme in the standard RF algorithm, and the decision is based on the majority. In contrast, this study replaces the voting scheme of the three trained RF classifiers with one trained using the multilayer perceptron (MLP) algorithm for decision making. The MLP-based classifier uses the aggregated outputs of the RF classifiers as new knowledge to train the ANN classifier to learn the hidden patterns that can collectively be extracted from the outputs of these three ensemble models. A detailed description of this is explained in the next section.

### 3.7. Phase 7: ANN Decision Making

In this phase, a multilayer perceptron (MLP) artificial neural network (ANN) classifier was constructed for decision making. The classifier was trained using a three-layer network consisting of 6 input neurons, 6 hidden neurons, and one output neuron. ANN has better generalization and can predict the actual class even with smaller data and complex nonlinear problems. Given a set of input features $X=\left(x_{1},\;x_{2},x_{3}\ldots x_{n}\right)$ and Y target class, the MLP learns the relationship between the X and Y. Some parameters affect the performance of the neural network such as weight initialization, biases, the activation function, the loss function, the optimizer, the number of hidden layers, and the number of neurons in each layer. The activation function provides output for the next layer by calculating the sum of the products of numerical values of input features by their weights. The loss or cost function is used to determine the classification error while the optimizer is used to reduce the error. In this study, the Broyden–Fletcher–Goldfarb–Shanno (BFGS) algorithm was used as the optimization algorithm. BFGS is a local search and gradient-based algorithm that is suitable for unconstrained nonlinear optimization problems to effectively determine the decent direction. It approximates the second derivative of the cost function (the Hessian) when the second derivative cannot be detected. The Sigmoid function in the following equation is used as an activation function:

(6) $$Segmoid\left(x\right)=\frac{1}{1+e^{-x}}$$

where x denotes the classification score and e is the natural logarithm which is approximately equal to 2.718281828.

**Figure 3:**
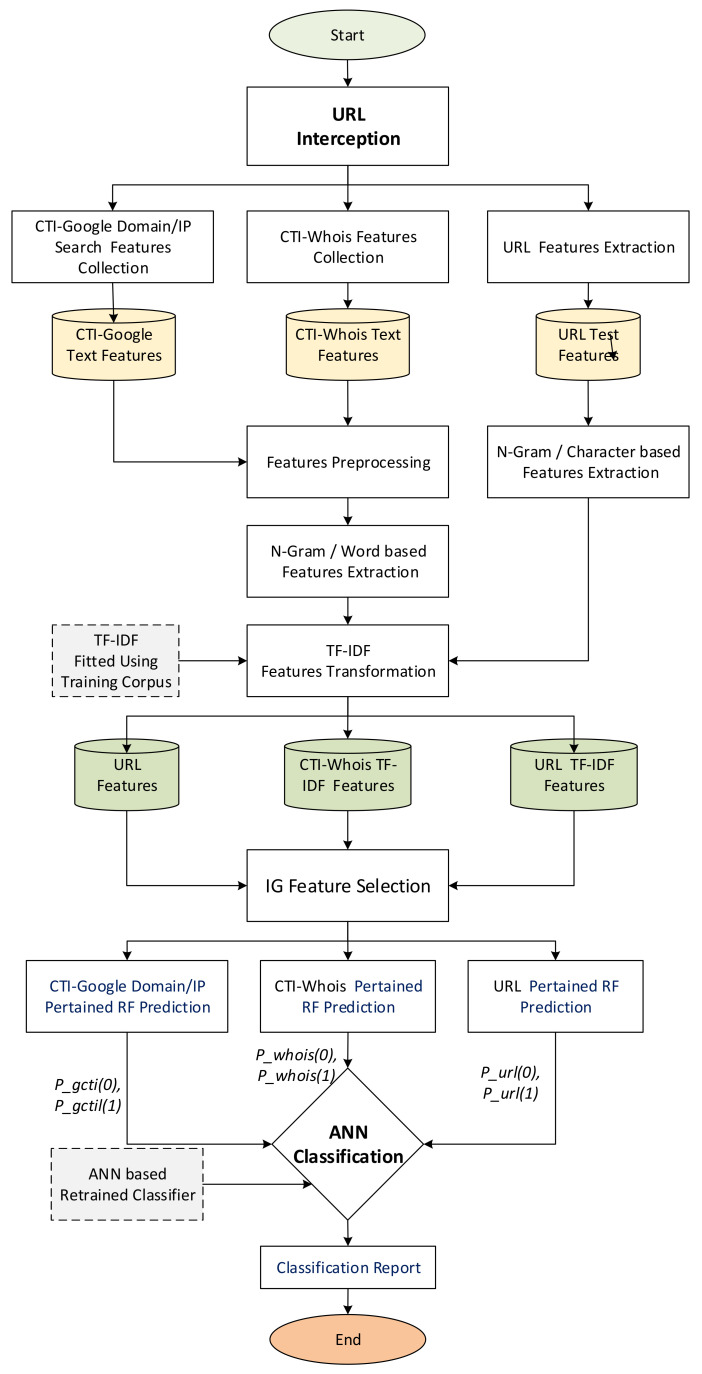
Flowchart of the CTI-MURLD model Operation.

To summarize, Figure 3 illustrates the operations of the proposed CTI-MURLD model. As can be seen in Figure 3, once the URL was intercepted (e.g., by the network sniffer of the detection system), three types of features were collected: the first types were the URL features such as the domain name, sub-domains, and types; the second types were the CTI features which were collected from a Google search; and the third types of features were collected from Whois information. These features were preprocessed, enriched using N-gram, and represented using TF-IDF techniques, as described in Section 3.1, Section 3.2 and Section 3.3. The important features were selected and input into the three pre-trained RF-based prediction models. Inspired by the divide and conquer principle, the RF prediction models were trained based on a single type of feature set, namely, CTI-Google, CTI-Whois, or URL features. The probabilistic aggregated outputs of the three RF prediction models (total output were 6 variables as shown in Figure 2, two values for each classifier) were used as input for the ANN-based classifier. The ANN classifier was used to learn the correlation between the RF prediction scores and the target class. It replaced the majority voting schemes used by the three RF classifiers for more accurate detection. Without this divide and conquer principle, such a correlation would not be released due to the curse of dimensionality because of the massive set of extracted multifaceted features.

## 4. Performance Evaluation

In this section, the used dataset, the experimental procedures, and the performance evaluation are described.

### 4.1. Sources and Preprocessing of Datasets

This study used a malicious URLs dataset that is publicly available on the Kaggle.com repository (available at https://www.kaggle.com/sid321axn/malicious-urls-dataset, accessed on 25 February 2022). The dataset was collected from widely-used sources by researchers of malicious URL detection domains such as Phishtank [39,40] (available at https://phishtank.org/, accessed on 25 February 2022) and URL dataset (ISCX-URL-2016) [8] (available at https://www.unb.ca/cic/datasets/url-2016.html, accessed on 25 February 2022). The URLs in the dataset were categorized into two types, malicious and benign. Malicious URLs included malware links, web defacement, spam, phishing, drive-by downloads, etc. A random sample consisting of 20,000 URLs was drawn and used in this study. Table 1 shows the number and types of URL samples in the datasets.

### 4.2. Experimental Procedures

The dataset was split into training and testing sets with 70% for training and 30% for testing. The training dataset was used to train the RF and MLP/ANN classifiers. The outputs (prediction values) of RF classes were used to train the ANN-based decision-making classifier. For each RF classifier, 100 estimators were created. For the ANN prediction model, the Broyden–Fletcher–Goldfarb–Shanno (BFGS) algorithm was used as the optimization algorithm. BFGS is one of the variants of the gradient descent algorithm and has proven to have better accuracy than the plain gradient descent algorithm. The learning rate was set close to zero i.e., 10–5 for better generalizability. Meanwhile, the logistic sigmoid function was used as an activation function. The neural network prediction model consisted of three layers, the input, hidden, and output layers. The input layer consisted of 6 neurons, the hidden layer contained 6 neurons, and the output layer contained a single neuron.

### 4.3. Performance Evaluation

To validate the detection performance of the proposed model, five performance measures were used: the overall accuracy; the detection rate (recall); the precision; the F1 score; the false-positive rate (FPR); and the false-negative rate (FNR). These performance measures are commonly used to evaluate the accuracy of the malware detection solutions in the literature. To evaluate the proposed model, the commonly used machine learning techniques that were used to evaluate the related malicious URL detection were used. Moreover, three models were developed for the evaluation of the CTI-MURLD, Google-CTI, Whois-CTI, and lexical URL-based features as baselines [8,11,13,14,15,23,41]. Furthermore, two deep learning-based models were developed for the evaluation of SDL and CNN-based malicious URL-detection models. A detailed description of the results is illustrated in the following section. The following equations were used for calculating the used performance measures in this study.

(7) $$Accuracy=\frac{TP+TN}{TP+TN+FP+FN}$$

(8) $$FPR=\frac{FP}{TP+FN}$$

(9) $$DR\;\left(Recall\right)=\frac{TP}{TP+FN}$$

(10) $$Precision=\frac{TP}{TP+FP}$$

(11) $$\text{F-measure}=\frac{2\times Precision\times Recall}{Precision+Recall}$$

For comparison, the base classifier of CTI-MURLD has been trained using state-of-the-art machine learning techniques including deep learning that have been used for malicious website detection, namely naïve Bayes (NB), logistic regression (LR), decision tree (DT), random forest (RF), convolutional neural network (CNN), and sequential deep learning (SDL) models.

## 5. Results and Discussion

**Figure 4:**
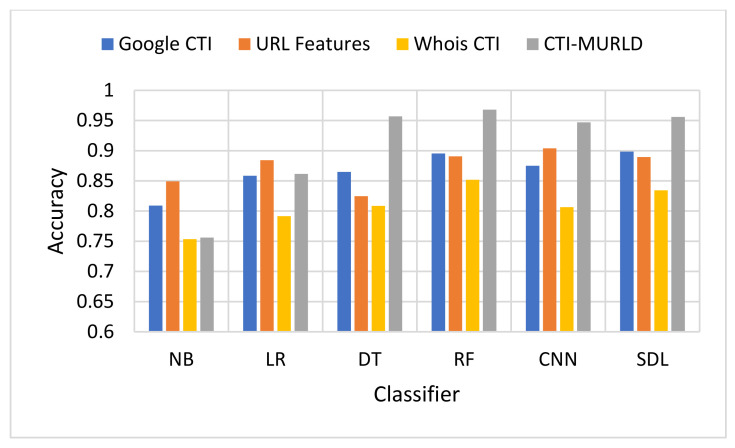
Comparison in terms of the detection-accuracy performance.

**Figure 5:**
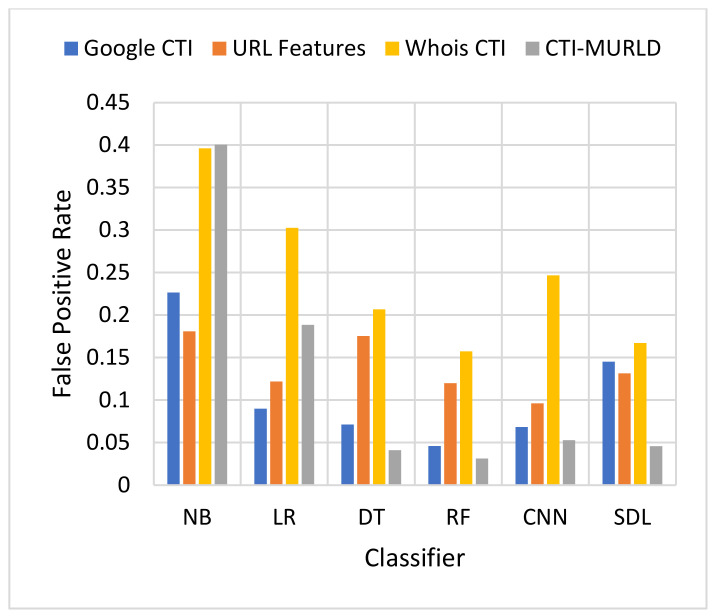
Comparison in terms of the FPR.

**Figure 6:**
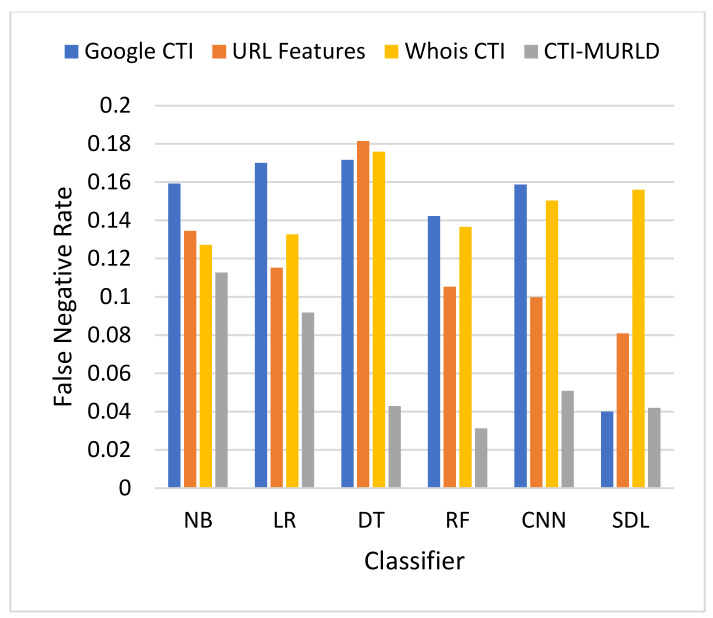
Comparison in terms of the FNR.

**Figure 7:**
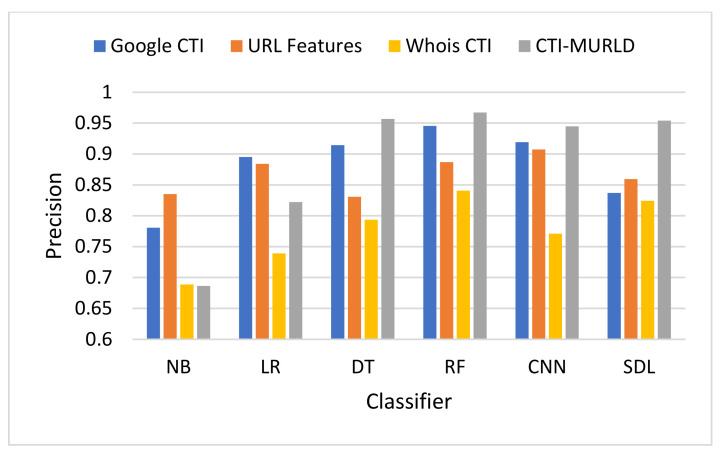
Comparison in terms of the precision.

**Figure 8:**
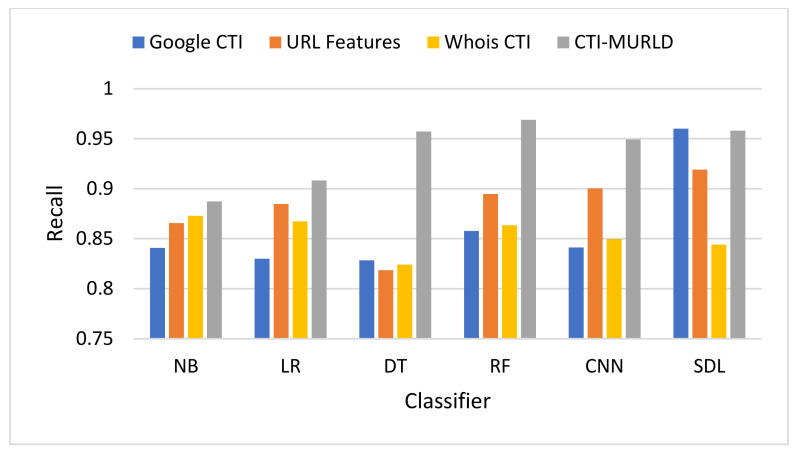
Comparison in terms of the recall (True Positive Rate).

**Figure 9:**
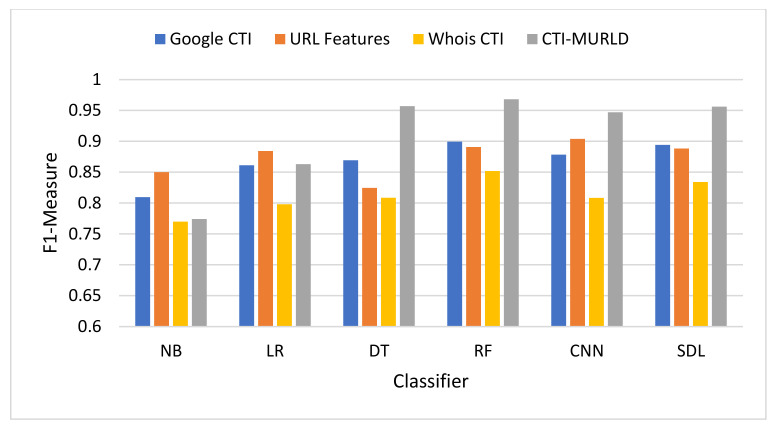
Comparison in terms of the F1-Measure.

The proposed Cyber Threat Intelligence-based Malicious URL Detection (CTI-MURLD) model has been validated using the aforementioned dataset and performance measures. Additionally, it was evaluated against the commonly used feature sets including the URL-based features and Whois-based features. Different feature sets have been compared to evaluate the proposed CTI-MURLD model. Table 2 and Figure 4, Figure 5, Figure 6, Figure 7, Figure 8 and Figure 9 illustrate the results obtained in terms of the detection accuracy, false-positive rate, false-negative rate, precision, recall, and F1-Measure, respectively.

Table 2 presents the performance of the CTI-MURLD model. As can be seen in Table 1, the RF technique outperformed the other machine learning algorithms. The decision tree algorithm also achieved the second-best performance while the sequential deep learning model and the CNN model performed slightly lower compared with RF and DT. However, the sequential model SDL model was better than the CNN model. It was expected that RF will perform better than DT because RF is a collection of DTs. A single DT can make a series of decisions based on the given set of features based on the information gained.

In terms of accuracy, Figure 4 depicts the accuracy performance of the proposed CTI-MURLD model compared with related works. In most cases, the CTI-based features especially the combined ones, outperformed the traditional-based features and the single set of features. For example, RF and DT DSL achieved accuracy higher than 95% with the combined CTI-based features. Meanwhile, the accuracy of the same classifiers using URL-based features was always lower than 90% except with the CNN model which achieved 90.8%. CNN was a commonly reported method that outperforms the conventional machine learning classifiers. However, it is believed that CNN performance depends on the competence of the representative features present in the image-like matrix. In the case of a huge number of features such as CTI-based features, a large portion of the dataset should be available to achieve maximum accuracy. It is worth mentioning that Whois-based features alone achieved the worst performance among the tested features while Google-based CTI achieved almost similar performance results to the URL-based features. For example, with the models DT, RF, SDL, LR, and NB, Google-based CTI features achieved the second-best accuracy performance. This indicates that the CTI-based features can complement the traditional features for detecting evasive malicious websites which try to evade detection by looking similar to benign websites.

Figure 5 and Figure 6 present the results in terms of FPR and FNR, respectively. The models designed using the combined CTI-based features and the Google-based CTI features achieved the lowest rate of false positives while Whois-based features achieved the highest rate of false positives. However, the combined-based CTI features achieved the lowest rate of false negatives. Meanwhile, the models designed based on the Google-based CTI features suffered from a high rate of false negatives. In general, all models designed with a single feature set such as the URL, Whois, or Google CTI suffered from a considerable number of false negatives. This is because it is difficult to differentiate between some malicious websites such as spoofing websites and other benign websites. Meanwhile, the proposed combined features set with the RF classifier achieved the lowest rate of false negatives which was 3.3% followed by SDL (4.2%) and DT (4.29%).

Figure 7 depicts the performance in terms of precision. The precision measures the predictability of the positive class. The proposed CTI-MURLD models using decision trees and deep learning-based classifiers achieved the highest precision compared with the URL-based features and the single set features such as Google CTI and Whois information. The proposed CTI-MURLD model achieved 96.7% precision using the RF classifier, 95.69% using the DT, 95.41% using SDL, and 94.48% using the CNN-based classifier. The models designed using the Google-based CTI features achieved the second-best results for precision. For instance, it achieved 94.54% using the RF-based classifier and 91.43% using the DT classifier. Meanwhile, the model’s design using Whois information is unprecise with all classifiers compared with the URL-based and the other CTI features.

Figure 8 shows the performance in terms of the recall or the defection rate. The proposed CTI-MURLD models using all classifiers outperformed the other types of feature sets, namely, URL-based features and single-set features such as Google CTI and Whois information. It achieved a 96.88% true positive rate using the RF-based classifier, 95.8% using the SDL, 95.71% using DT, 94.91% using the CNN, 90.82%, and 88.73% using NB-based classifier. The models designed using other feature sets vary lower than 90% except with SDL-based classifier, the Google-based CTI features achieve 96% and URL-based features achieve 92%.

The harmonic means results in Figure 9 in terms of F-Measure (also called F1-Score) summarizes how well the model performs with both precision and recall. The proposed CTI-MWD model using a decision tree and deep learning-based classifiers achieved the highest F1-Score value compared with the URL-based features and the single set features such as Google CTI and Whois information. In most cases, the F1-Score of the proposed CTI-MWD model achieves a 95% or higher value. It achieves a 96.8% score using the RF classifier, 95.7% using the DT, 95.61% using SDL, and 94.69% using the CNN-based classifier. The models designed using other feature sets vary lower than 90% except with CN-based classifier and the Whois information-based features the model achieves 90% and the model designed using Google-based CTI features with RF archives 90% F-Score.

As RF implicit feature selection by applying information gain and feature importance, an experiment was conducted to evaluate the effectiveness of the feature selection used by the model before applying the RF algorithms. Given that the datasets used in this study were text data containing a massive number of features, most of these features were irrelevant and should be eliminated. The results in Table 3 (see RF without FS in Table 3) indicate that the RF with the proposed feature selection achieved better than the RF without the selection. The RF randomly selects a subset feature to train each weak classifier. The selection of the important features happened within the subset. In contrast, in this study, selecting the importance classifiers before the RF enforces the RF algorithm to select the subset features of the pool of the selected important features, which improves the accuracy of the weak classifier and thus the overall accuracy.

Because RF algorithms use a different range of hyperparameters, a grid search is used to search for the best parameters that improve the performance. Table 3 (see RF with GS in Table 3) shows the performance results of the grid search. Based on the grid search results, the hyperparameters that gave the best performance were: 1000 estimators, five minimum sample split, two minimum samples leaf, an unlimited number of leaf nodes, samples drawn with replacement, and an unlimited maximum number of features. As shown in Table 3, the performance was further improved, as expected.

The results obtained using the proposed CTI-MURLD model raise an interesting but fundamental question of why the ensemble-based RF and DT-based classifiers achieved the best results compared with the deep learning-based classifiers. The answer lies in the dataset itself, the number of features extracted using the Google-based CTI was huge compared to the URL or the Whois information features. This created highly noisy data with a sparse feature vector. Moreover, when the features were combined from CTI, Whois information, and URL-based features a high-dimensional problem was created. In this situation, DT and RF were suitable for high-dimensional noisy data [42]. With such high-dimensional features, deep learning models such as CNN and SDL need a larger dataset to attain their maximum performance. In addition, neural network-based classifiers create patterns by connecting neurons with each other which is difficult to generalize in high-dimensional datasets. Meanwhile, the RF classifier creates independent patterns which are suitable for high-dimensional data and small datasets. This may be an indication of why the decision tree-based classifier outperformed the deep learning model.

## 6. Conclusions

In this study, a malicious website detection model was designed and developed based on cyber threat intelligence extracted from Google. The first main contribution of these studies was the use of cyber threat intelligence as a new set of features with a hypothesis stating that cyber threat intelligence is an effective and safer alternative to improve the detection accuracy of malicious websites. The domain names of the websites were extracted using the Whois technique, and cyber threat intelligence was collected from Google and combined with URL-based features. Due to the diversity of attack vectors of malicious websites, high-dimensional features were created and used to train the proposed model. The second main contribution of this study is in the design of the proposed detection model. Three random forest classifiers were developed, each of which was trained based on different features, namely, URL-based features, cyber threat intelligence features based on Google, and Whois information-based features. The probabilistic outputs of the weak classifiers in each tree were aggregated and used as input features to a multilayer perceptron designed to replace the three majority voting schemes used by the trained random forest classifiers. Several types of machine learning classifiers were investigated to validate and evaluate the proposed model. Results show that the CTI-based features significantly improved the detection performance, achieving 96.80% compared with the best 90.4% achieved by the URL-based features. The false-positive rate was significantly decreased to 3.1% compared with 12% performed by the URL-based model. The main drawback of this study is that the cyber threat intelligence collected is obtained from a Google search. Such a source of data is not necessarily reliable and hence, false information is highly probable. As a result, a solution based on a trusted source could be a possible future direction for other researchers.

## Figures and Tables

**Table 1:** Number and types of URLs used in this study.

Category	Number of Samples
Total URLs	651,191
Total Benign	428,103
Total Malicious	223,088
Malicious URLs	
Defacement	96,457
Phishing	94,111
Malware Link	32,520

**Table 2:** Performance of the CTI-MURLD model using Different Classifiers.

Ensemble Classifiers	Accuracy	FPR	FNR	Recall	Precession	F1
NB	75.60%	40.04%	11.27%	88.73%	68.65%	77.41%
LR	86.15%	18.85%	9.18%	90.82%	82.21%	86.30%
DT	95.70%	4.10%	4.29%	95.71%	95.69%	95.70%
RF	96.80%	3.13%	3.13%	96.88%	96.72%	96.80%
CNN	94.70%	5.27%	5.09%	94.91%	94.48%	94.69%
SDL	95.61%	4.57%	4.20%	95.80%	95.41%	95.61%

**Table 3:** Performance of the CTI-MURLD model with and without feature selection and with grid search best-found hyperparameters.

Ensemble Classifiers	Accuracy	FPR	FNR	Recall	Precession	F1
RF without FS	96.30%	3.81%	3.59%	96.20%	96.58%	96.39%
RF with FS	96.80%	3.13%	3.13%	96.88%	96.72%	96.80%
RF with GS	97.25%	2.73%	2.76%	97.26%	97.36%	97.31%

## Data Availability

The dataset that is used in this study is publicly available on the Kaggle.com repository (https://www.kaggle.com/sid321axn/malicious-urls-dataset, last access 25 February 2022).
